# Humanized Anti-MUC16 Antibody-Conjugated Contrast Agents for Magnetic Resonance Imaging of Pancreatic Cancer

**DOI:** 10.3390/cancers17060957

**Published:** 2025-03-12

**Authors:** Jayasindu Mathiyazhagan, Christabelle Rajesh, Satish Sagar, Thomas C. Caffrey, Ying Huang, Aaron M. Mohs, Benjamin J. Swanson, Michael A. Hollingsworth, Cory L. Brooks, Prakash Radhakrishnan

**Affiliations:** 1 Eppley Institute for Research in Cancer and Allied Diseases, University of Nebraska Medical Center, Omaha, NE 68198, USA; 2 Fred & Pamela Buffett Cancer Center, University of Nebraska Medical Center, Omaha, NE 68198, USA; 3 Department of Pharmaceutical Sciences, University of Nebraska Medical Center, Omaha, NE 68198, USA; 4 Department of Pathology and Microbiology, University of Nebraska Medical Center, Omaha, NE 68198, USA; 5 Department of Chemistry and Biochemistry, California State University Fresno, Fresno, CA 93740, USA

**Keywords:** MUC16, pancreatic cancer, magnetic resonance imaging, early detection

## Abstract

**Background/Objectives:** Pancreatic ductal adenocarcinoma (PDAC) is diagnosed at a late stage with distant metastasis in an overwhelming 50% of cases, and the prognosis is poor. Treating this extremely aggressive disease with standard-of-care therapies has led to modest benefits in overall survival, mainly due to a lack of targeted early treatment modalities, as early detection has not yet been possible. Mucin-16 (MUC16) is a glycoprotein overexpressed in more than 60% of patients with PDAC and is a tumor-specific biomarker. **Methods:** In this study, a magnetic resonance imaging (MRI) probe to facilitate the detection of early and late lesions of PDAC is developed by conjugating a MUC16-targeted humanized antibody (huAR9.6) with gadolinium. **Results:** In preclinical mouse models, this MUC16-targeted MRI probe demonstrates effective contrast enhancement in early lesions of PDAC in the subcutaneous setting and allows for the detection of late-stage pancreatic cancer tumors in an orthotopic model. The probe did not induce any toxicity in vital organs at the administered doses. **Conclusions:** This study establishes that synthesizing a MUC16-targeted MRI probe is feasible and allows for the better high-resolution contrast enhancement of MUC16+ PDAC lesions to facilitate detection and possibly better treatment strategies.

## Simple Summary

This study demonstrates the synthesis of a MUC16-targeted antibody–gadolinium magnetic resonance imaging (MRI) conjugate for pancreatic cancer. The purpose of this conjugate is to help with detection via the high-resolution MR imaging of pancreatic cancer lesions, which often go unchecked and can become aggressive and recalcitrant to therapy. The MUC16-targeted conjugate facilitates cancer detection in early- and late-stage disease models over 24 h post-administration and does not induce toxicity even after 96 h in the circulation. This humanized MRI probe can potentially advance the detection of pancreatic cancer in the clinic.

## 1. Introduction

Pancreatic ductal adenocarcinoma (PDAC) is a lethal malignancy with a dismal prognosis, evidenced by its low 5-year overall survival post-diagnosis of 13% [1,2]. PDAC is often diagnosed at a late stage, with patients presenting with unresectable disease owing to distant metastases, during which standard-of-care chemotherapy has marginal benefits [3]. This is partly due to the challenges faced in developing detection tools, particularly for the early diagnosis of pancreatic cancer. Magnetic resonance imaging (MRI) is part of a routine diagnostic methodology used to detect a pancreatic cancer lesion or tumor masses, as the technology has become sophisticated over the years, providing high-resolution scans for better detection [4]. It has been postulated that early diagnosis of this therapy for recalcitrant disease could provide favorable patient outcomes, leading to a higher quality of life. MRI can provide clinicians with high-resolution anatomical images that are non-invasive and radiation-free. More specifically, MRI has higher spatial (1–2 mm) and temporal resolution (20–50 milliseconds) than other commonly used imaging modalities like positron imaging tomography (PET) [5]. Using contrast-enhancing tools like gadolinium (Gd^3+^) has further enabled the robust detection of relatively smaller-sized tumor lesions, adding to the benefits of high spatial resolution with MRI [6]. Many contrast agents, including Gd^3+^ complexes, enhance regions of accumulation indicative of disease. In the context of cancer, this facilitates identifying tumors (or tumor regions). Gd^3+^ requires a ligand such as diethylenetriaminepentaacetic (DTPA) dianhydride to form a stable conjugate that prevents the release of free Gd^3+^, which is toxic [7,8,9]. However, a crucial drawback of using MRI in cancer diagnoses involves the potentially inaccurate distinguishment of cancer tissue from normal tissue due to the accumulation of unconjugated gadolinium-based contrast agents in non-cancerous tissue [10].

Targeted imaging using cancer biomarkers is a method that has been studied for several decades to aid in detecting cancer lesions [11,12,13,14,15,16]. A study demonstrated that a peptide probe conjugated to gadolinium was able to bind fibrin/fibronectin complexes found in the tumors of patients with breast cancer. This MRI peptide probe facilitates the contrast enhancement of micrometastatic lesions, which can also aid in the early detection of this aggressive disease [17]. Another study used ICAM1 as a pancreatic cancer biomarker and showed that an anti-ICAM1 antibody MRI proves to aid in the detection of aggressive and large pancreatic cancer lesions in mouse models [9]. Epitope CA125 in Mucin-16 (MUC16) is a well-known diagnostic marker of various cancers, including ovarian and pancreas cancer [18,19,20,21,22]. In normal physiological conditions, heavily glycosylated proteins like mucins protect epithelial cells by creating a physical barrier against various damage-inducing stressors in the microenvironment [23,24]. Cancer-associated mucins exhibit altered glycosylation that promotes oncogenic events during the progression of different diseases, including pancreatic cancer [25,26,27,28]. MUC16 is overexpressed in more than 65% of patients with PDAC, is heavily involved in facilitating the progression of this disease, and is virtually absent in the normal pancreas, making it a robust tumor-associated antigen that can be used to detect cancerous lesions in the pancreas [18,22]. In the last half-decade, MUC16 has been used for the biomarker-based detection of cancer using tools like fluorescence-guided surgery (FGS) and positron emission tomography (PET) imaging [13,29,30]. AR9.6 is a monoclonal antibody that binds to a sea urchin, Enterokinase, and Agrin (SEA) domain 5 in the C-terminal extracellular region of MUC. The binding of AR9.6 to SEA5 of MUC16 interferes with MUC16′s interaction with cell surface receptors of the ErbB family and their downstream signaling pathways through AKT and GSK3β, eventually disrupting cancer growth [22].

In this study, we describe the development of a MUC16-targeted antibody–MRI conjugate for detecting pancreatic cancer at different stages in preclinical models. We use in vitro models, as well as in vivo subcutaneous (early-stage) and orthotopic (late-stage) models, to demonstrate the efficacy of humanized AR9.6 (huAR9.6) conjugated to Gd-DTPA to detect PDAC lesions and employ this humanized antibody MRI probe to facilitate the detection of MUC16-expressing PDAC lesions in patients.

## 2. Methods

### 2.1. Conjugate Preparation

The huAR9.6-DTPA-Gd conjugate and huIgG-DTPA-Gd (isotype-matched negative control) were prepared by adding 0.2 mg of DTPA dianhydride to equal amounts of antibodies, namely huAR9.6 or huIgG in NaHCO_3_ buffer (0.1 M, pH 9.0). The above solution was rotated overnight at room temperature, and the following day, the solution was filtered to remove unbound DTPA using a 30 K MWCO centrifugal filter. The concentrate was redispersed in Citrate Buffer (0.1 M, pH 6.5), and then 0.1 mg of gadolinium chloride (GdCl_3_·6H_2_O) was mixed with the above DTPA–antibody mixture in rotation for 24 h at room temperature. Further, the free Gd^3+^ was removed by a 30 K MWCO centrifugal filter, and the concentrated conjugate (huIgG–Gd–DTPA and huAR9.6-Gd-DTPA) was redispersed in PBS for further analysis and use. As the conjugation of GdCl_3_ to DTPA acidifies the solution due to the release of protons, synthesis of the above conjugates using Gd-acetate was also performed. This acidification can affect the efficiency and stability of a conjugate. In contrast, Gd-acetate acts as a buffer, neutralizing the released protons and maintaining a more stable pH.

### 2.2. Characterization of the Conjugate

The Gd^3+^ concentrations in huIgG–Gd–DTPA and huAR9.6–Gd–DTPA were analyzed by ICP–MS (Inductively Coupled Plasma–Mass Spectrometry). The conjugate antibody concentrations were measured using absorbance via Nanodrop (NanoDrop One, Thermo Scientific, Waltham, MA, USA). The antibody to Gd ratio was calculated based on the antibody and Gd concentration in the conjugates. Free Gd^3+^ in the concentrate was analyzed using an xylenol orange assay. An amount of 0.15 mg of xylenol orange (XO) was dissolved in 10 mL acetate buffer (pH 5.8). Various concentrations of Gd^3+^ (0, 1, 2, 3, 4, and 5 μM) were dissolved in acetate buffer for the standard curve. Finally, 100 μL of Gd^3+^ was added to 900 μL of XO and mixed vigorously. The Gd standards and conjugates were then measured using UV-VIS spectrometry (Evolution 220 UV-visible spectrophotometer, Thermo Scientific, Waltham, MA, USA) at 300 nm–800 nm wavelengths. The samples (huIgG–Gd–DTPA and huAR9.6-Gd-DTPA) were measured for free Gd^3+^ content by interpolation from the standard curve.

### 2.3. Interaction and Competitive Binding of huAR9.6 with MUC16

The binding of the monoclonal antibody huAR9.6 to MUC16 was validated in cell lines by immunofluorescence (IF) staining, as described previously [9]. T3 M4 wildtype (WT) and T3 M4 MUC16 ^KO^ cells were cultured and fixed in 4% paraformaldehyde following permeabilization with 0.15% Triton X. The cells were incubated with huAR9.6 mAb at room temperature for 2 h. The cells were washed with phosphate-buffer saline (PBS) and incubated with the Alexa Fluor-647 conjugated donkey anti-human IgG secondary antibody (Jackson ImmunoResearch, West Grove, PA, USA). Following incubation with a secondary antibody, the cells were washed and mounted using a Vectashield mounting medium containing 4′,6-diamidino-2-phenylindole (DAPI) (Vector Laboratories, Burlingame, CA, USA). The slides were viewed under confocal laser scanning microscopy at the UNMC Core Facility.

Additionally, an ELISA (Enzyme-linked immunosorbent assay) was performed as follows: a recombinant MUC16 fragment (Trx-1.2 T, 20 μg/mL in PBS) was immobilized in the wells of MaxiSorp microtiter plates (Thermo Fisher Scientific, Waltham, MA, USA) and blocked overnight (4 °C) with bovine serum albumin (1% in PBS) as previously described (19). Conjugated or unconjugated huAR9.6 antibody was serially diluted and applied to the plate. The binding of huAR9.6 was detected by adding 1:40,000 diluted horseradish peroxidase (HRP)-conjugated goat anti-human kappa IgG (Novus Biologicals, Centennial, CO, USA). For signal development, 1-Step Ultra TMB substrate (Thermo Fisher Scientific) was added, and the reaction was stopped with 0.18 M H_2_SO_4_. Absorbances at 450 and 540 nm were measured and subtracted (450–540 nm) using a 96-well plate reader (Molecular Devices SpectraMax iD3, Molecular Devices, San Josa, CA, USA); blanks were subtracted. The absorbance was plotted against the log concentration of the antibody. Data were fitted using a 4-parameter logistic curve, and *EC*50 values were determined from the fit. All experiments were performed in triplicate.

### 2.4. In Vitro MR Imaging of Pancreatic Cancer Cell Lines

The MUC16+ T3 M4 cell line was used for the study. The cell line was maintained in Dulbecco’s modified Eagle’s medium (DMEM) supplemented with 10% fetal bovine serum (FBS) and 1% antibiotic solution (100 units/mL penicillin and 100 mg/mL streptomycin) and grown in a humidified incubator with 5% CO_2_ at 37 °C. To assess the interaction of the huIgG–Gd–DTPA and huAR9.6–Gd–DTPA conjugates with MUC16+ T3 M4 pancreatic cancer cell lines, the cell lines were incubated with the conjugates at various concentrations of Gd (150 nM, 300 nM, and 600 nM). After 4 h of incubation, the cell lines were subjected to MR imaging. A second round of this experiment was performed with a 450 nM Gd concentration. MR imaging was performed with a Tesla MRI scanner (Bruker BioSpec 70/20, Billerica, MA.; MRI Core Facility at UNMC) with Turbo spin-echo sequence for T2-weighted MRI.

### 2.5. In Vivo Subcutaneous PDAC Mouse Model and MR Imaging

All animal studies were conducted per the Institutional Animal Care and Use Committee (IACUC) at UNMC. The MUC16+ T3 M4 cell line was injected subcutaneously into 6–8 week-old athymic nude mice (20–25 g, NCr-nu/nu, Strain 01 B74, Charles River, Wilmington, MA, USA). After 11 days of tumor cell implantation, the animals were divided into two groups (*n* = 3; each) and injected with huIgG–Gd–DTPA or huAR9.6–Gd–DTPA intravenously (50 nmol/gram body weight). MR imaging was performed at pre-injection and then at 24, 48, 72, and 96 h post-injection with a Tesla MRI, with a Turbo spin-echo sequence for T1- and T2-weighted MRI, as gadolinium-based contrast agents can affect T1-weighted images, increasing the signal intensity by shortening the relaxation time, while they have minimal effects on T2-weighted images. The signal intensity and the ROI in tumors were drawn around the whole tumor at the same slice with the same imaging depth upon consultation with the small animal MRI Core Facility at UNMC. ImageJ software (version 8) calculated and normalized the pixel intensities to the area of ROIs.

### 2.6. In Vivo Orthotopic PDAC Mouse Model and MR Imaging

All animal studies were conducted per the Institutional Animal Care and Use Committee (IACUC) at UNMC. The mice were orthotopically implanted with the MUC16+ T3 M4 cell line into the pancreas of 6–8-week-old athymic mice (20–25 g, NCr-nu/nu, Strain 01 B74, Charles River). After 14 days of tumor development, the animals (Female, *n* = 6/group) were injected with huIgG–Gd–DTPA or huAR9.6–Gd–DTPA intravenously (100 nmol/gram body weight). MR imaging was performed at pre-injection and then 3, 24 and 144 h post-injection with a Tesla MRI scanner, with a Turbo spin-echo sequence for T1- and T2-weighted MRI. The signal intensity and the ROI in tumors were drawn around the whole tumor at the same slice with the same imaging depth. The pixel intensities were calculated and normalized to the area of ROIs by ImageJ software.

### 2.7. Biochemical Analysis and Histology

Since Gd^3+^ is a highly toxic metal, we evaluated antibody–Gd conjugate or released Gd-induced biochemical changes in the blood using the levels observed by analyzing alanine transaminase (ALT) and aspartate transaminase (AST) as a measure of liver function to assess conjugate-induced liver toxicity. Histological alterations in vital organs (liver, lung, kidney, and spleen) were evaluated by a pathologist. The blood collected 24 h post-injection was subjected to ICP–MS analysis to measure the circulating free Gd content.

### 2.8. Statistical Analysis

For all data collected from in vitro and in vivo studies (subcutaneous and orthotopic models), the results of the analyses are represented as mean ± standard error mean (SEM), and the significance was evaluated by two-way analysis of variance (ANOVA) with Tukey’s multiple comparison test. GraphPad Prism v10 was used to prepare representative graphs.

## 3. Results

### 3.1. Characterization of the MUC16 Targeted huAR9.6 MRI Conjugate

**Figure 1:**
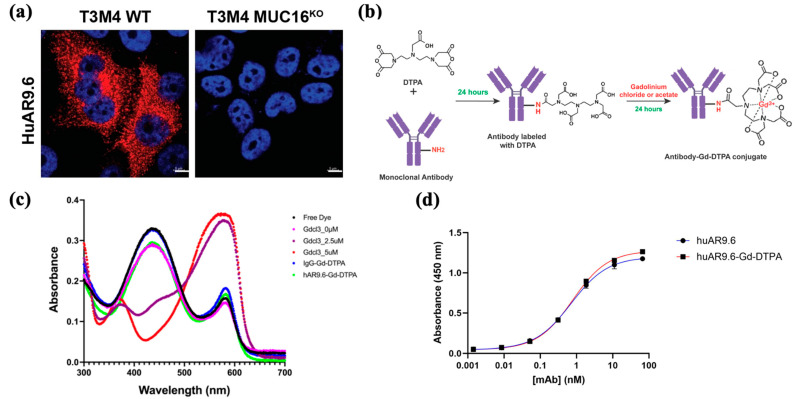
Targeting MRI conjugate. (**a**) Immunofluorescence (IF) staining for MUC16 using huAR9.6 on T3 M4 WT (MUC16+) and T3 M4 MUC16 ^KO^ isogenic PDAC cell lines; (**b**) synthesis of the antibody conjugate for MRI via conjugation of huAR9.6 and huIgG (isotype control) with DTPA and gadolinium chloride to yield antibody–Gd–DTPA; (**c**) the spectrometric distribution (300 nm to 700 nm) of standard Gd chloride, huIgG–Gd-DTPA and huAR9.6–Gd–DTPA conjugates; (**d**) enzyme-linked immunosorbent assay (ELIZA) depicting huAR9.6 and huAR9.6–Gd–DTPA binding affinity to MUC16 TR 1.2 (*n* = 3).

To assess huAR9.6 as a potential diagnostic agent for MUC16-expressing PDAC, we confirmed that huAR9.6 binds to MUC16-expressing PDAC cells, T3M4 and that does not bind to MUC16^KO^ cells using immunofluorescence. (Figure 1a). This validated the specificity of this monoclonal antibody for MUC16 on PDAC cells. Synthesis of the antibody MRI conjugate was first performed using a sequential reaction to conjugate huAR9.6 or huIgG (isotype control) to diethylenetriamine pentaacetate dianhydride (DTPA dianhydride) for 24 h followed by conjugation to gadolinium using gadolinium chloride (GdCl_3_) for 24 h (Figure 1b) to yield an antibody–Gd–DTPA conjugate.

The pH of the conjugate with GdCl_3_ (formulated using the NaHCO_3_ and citrate buffers) was between 5 and 6. ICP-MS was conducted to determine the gadolinium concentration in the synthesized conjugates, and the Gd/Antibody ratio in huIgG–Gd–DTPA and huAR9.6–Gd–DTPA was found to be 54.81 and 22.61, respectively. Free Gd levels determined by xylenol orange assay for huIgG–Gd–DTPA and huAR9.6–Gd–DTPA were 0.28 µM and 0.29 µM, respectively, from 6360 µM of total Gd in the solution (Figure 1c). These amounts are negligible in terms of indicating any off-target or adverse effects, and measurement of this parameter is crucial when designing a non-toxic conjugate.

ELISA analysis revealed that the huAR9.6–Gd–DTPA conjugate showed comparable MUC16 TR1.2 binding affinity to the unconjugated antibody (Figure 1d), which suggests that Gd–DTPA conjugation did not affect the binding affinity of huAR9.6 to MUC16.

### 3.2. In Vitro Assessment of the MR Imaging Potential of huAR9.6-Gd-DTPA

**Figure 2:**
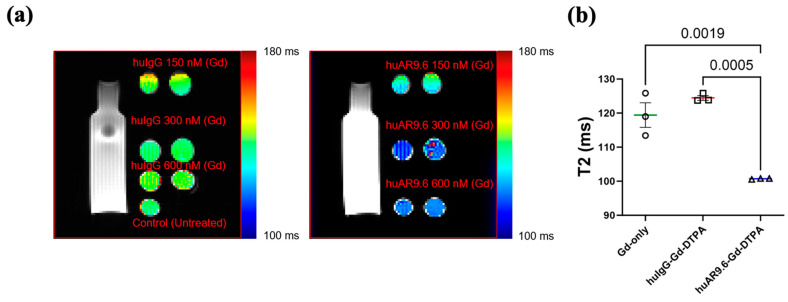
In vitro studies conducted to characterize the MRI conjugate: MR scans of T3 M4 cells treated with contrast agents and embedded in agar, (**a**) T2-weighted MRI of huIgG–Gd–DTPA and huAR9.6–Gd–DTPA at 150 nM, 300 nM, and 600 nM gadolinium; (**b**) graph depicting the T2 relaxation time (milliseconds) of gadolinium only (Magnevist), huIgG–Gd–DTPA and huAR9.6–Gd–DTPA (*n* = 3), all at a 450 nM gadolinium concentration. *p* < 0.05 is considered statistically significant.

After the antibody MRI conjugate was characterized, we conducted an in vitro assessment of its MR imaging potential. MUC16-expressing T3 M4 cells were incubated with the huIgG– and huAR9.6–Gd–DTPA conjugates at gadolinium concentrations of 150 nM, 300 nM, and 600 nM for 4 h and embedded in agar. T2-weighted images were processed, and the relaxation time in milliseconds (ms) was calculated (Figure 2a). Magnevist (Gadopentetic acid) was used as the Gd-only control. Based on this, a Gd concentration between 300 and 600 nM was deemed appropriate. Hence, we repeated the experiment, and T3 M4 cells incubated with huAR9.6–Gd–DTPA (Gd concentration 450 nM) had significantly lower relaxation times in all replicates when compared to comparable concentrations of the controls huIgG–Gd–DTPA and Gadopentetic acid (Figure 2b). This shorter relaxation time in the in vitro assay strongly suggests that MUC16-targeted huAR9.6–Gd–DTPA could aid in detecting MUC16-bearing pancreatic cancer cells and lesions.

### 3.3. huAR9.6–Gd–DTPA Successfully Detects Early Tumors in a Subcutaneous Model of Pancreatic Cancer

**Figure 3:**
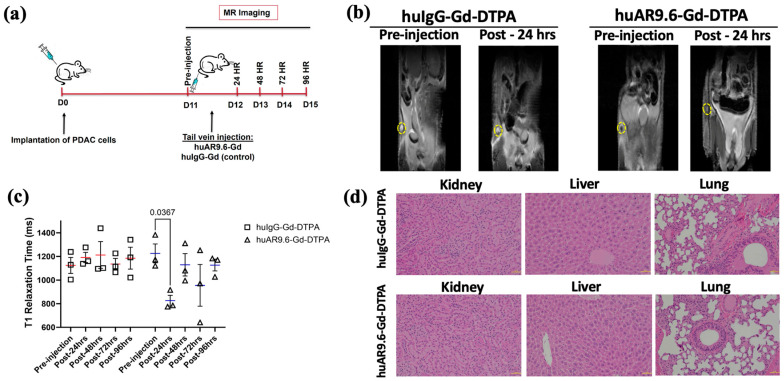
Assessment of antibody MR conjugate in a subcutaneous model of PDAC. (**a**) Schema of MRI of subcutaneous pancreatic cancer model injected with T3 M4 cells (1 × 10^6^ cells) at day 0 (D0), administered (intravenous, tail vein) with huAR9.6–Gd–DTPA or huIgG–Gd–DTPA (50 nM Gd/gram body weight) at 11 days post-tumor inoculation (*n* = 3/group) with MRI performed pre-injection and after 24, 48, 72 and 96 h; (**b**) T1 maps from in vivo subcutaneous tumor MRI of huIgG–Gd–DTPA and huAR9.6–Gd–DTPA groups treated pre-injection and post-24 h, subcutaneous tumors are circled in yellow; (**c**) the T1 relaxation time (milliseconds) of mice administered with huIgG–Gd–DTPA and huAR9.6–Gd–DTPA at various time points; (**d**) H&E staining of kidney, liver and lung tissues to assess morphological changes due to Gd toxicity (20× magnification). *p* < 0.05 is considered statistically significant.

As the huAR9.6–Gd–DTPA conjugate made using GdCl_3_ had thus far shown promising results, we conducted an early detection study for T3 M4-derived tumors that were smaller than ~35 mg (equivalent to <1 cm longitudinal lesions) and that started to be palpable in a subcutaneous model using athymic nude mice. As early detection is an important goal of our study, upon tumor palpation at 11 days post-implantation of T3 M4 cells, a pre-injection T1-weighted MR scan was performed. After this, mice were administered the huIgG– and huAR9.6–Gd–DTPA conjugates (i.v., tail vein) at a 50 nM Gd/gram body weight. Subsequent MR scans were conducted at 24, 48, 72 and 96 h post-injection (Figure 3a). The MRI signal (using T1 maps) was quantitatively measured using ImageJ for all the time points by locating and drawing the region of interest (ROI) around the tumor (yellow circles in Figure 3b). We observed a significant reduction in T1 relaxation times in tumors from huAR9.6–Gd–DTPA administered animals compared to huIgG–Gd–DTPA controls (Figure 3b,c). Such a decrease in T1 relaxation and contrast enhancement (beyond the effect rendered by gadolinium alone) was lost sometime after the first 24 h post-administration of the huAR9.6–Gd–DTPA conjugate (Figure 3c). No contrast enhancement was observed in any of the mice administered huIgG–Gd–DTPA, suggesting that huAR9.6 mediated that detection of MUC16-expressing cells within the tumor in the huAR9.6–Gd–DTPA cohort (Figure 3c).

Mice from both groups were euthanized at the experimental endpoint (day 15 post-tumor cell implantation and 96 h post-antibody conjugate administration). Vital organs were collected for histology analysis. No apparent histological alterations or signs of conjugate-induced toxicity were detected in mice’s kidneys, liver, or lungs in either of the two cohorts (Figure 3d). These results suggest that at the doses administered in this subcutaneous model of pancreatic cancer, the huAR9.6–Gd–DTPA conjugate enabled relatively early detection of MUC16-expressing pancreatic tumor lesions.

### 3.4. huAR9.6–Gd–DTPA Provides Contrast Enhancement for the Detection of In Vivo Orthotopic PDAC Tumors

Next, we tested the huAR9.6–Gd–DTPA conjugate in an anatomically accurate orthotopic model that better recapitulates the vasculature inside the tumor. huAR9.6 and huIgG were conjugated with Gd Acetate–DTPA for the in vivo orthotopic model to facilitate increased stability and less free Gd+ in the solution (Figure 1b).

**Figure 4:**
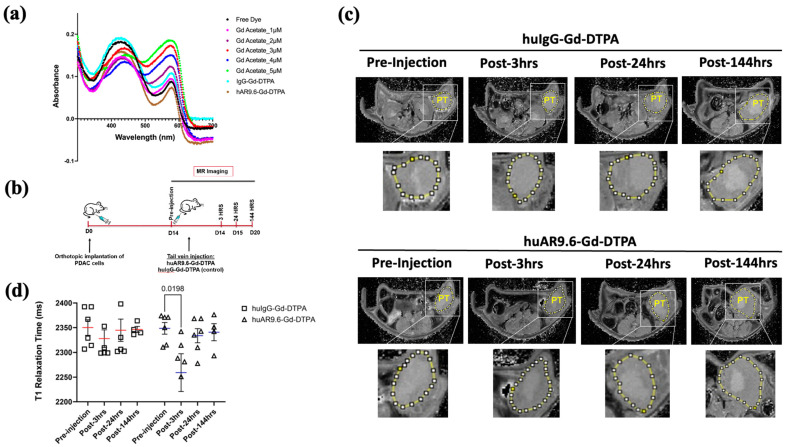
huAR9.6 gadolinium acetate facilitates MRI-based detection of pancreatic tumor in an orthotopic mouse model. (**a**) The spectrometric distribution (300 nm to 700 nm) of standard Gd –Acetate, huIgG–Gd–DTPA and huAR9.6–Gd–DTPA conjugates at varying concentrations; (**b**) Schema of the MRI of the in vivo orthotopic pancreatic cancer model bearing T3 M4 cell-derived tumors at day 0, pre-injection and 3, 24 and 144 h post-intravenous (tail vein) administration on day 14 with huAR9.6–Gd–DTPA or huIgG–Gd–DTPA (100 nM Gd/gram body weight, *n* = 6/group); (**c**) T1 imaging of huIgG–Gd–DTPA- and huAR9.6–Gd–DTPA-treated animals pre-injection, and 3 h, 24 h and 144 h post-conjugate injection. Pancreatic tumor annotated as PT and circled in yellow; (**d**) comparison of T1 relaxation times (milliseconds) of huIgG–Gd–DTPA and huAR9.6–Gd–DTPA at various time points. *p* < 0.05 is considered statistically significant. [PT.; pancreatic tumor].

The pH of the conjugate with Gd Acetate (synthesized entirely in NaHCO_3_ buffer) was between 7 and 8. A previous report implied that free Gd increases with decreasing pH, and Gd–DTPA conjugates are more stable in near-alkaline storage conditions [7]. Hence, we used these new huAR9.6–Gd–DTPA and control conjugates synthesized using Gd–acetate and NaHCO_3_ to achieve higher stability for administration into the orthotopic model. A 20:1 Gd-DTPA to antibody conjugation ratio has been optimal for antibody-based gadolinium contrast MRI agents [8,9]. The Gd/Antibody ratios in huIgG–Gd Acetate–DTPA and huAR9.6–Gd Acetate–DTPA were 28.39 (±0.06) and 31.32 (±0.31), respectively. The xylenol orange assay showed that the free Gd in the huIgG–Gd Acetate–DTPA and huAR9.6–Gd Acetate–DTPA were 0.60 µM and 0.058 µM, respectively, from 6360 µM of total Gd in the solution (Figure 4a).

MR imaging was performed in orthotopic tumor-bearing athymic nude mice pre-injection, and 3, 24, and 144 h post-conjugate injection (Figure 4b).

T1 relaxation was measured by drawing the region of interest (yellow circle in Figure 4c) and quantifying values using ImageJ for T1 maps for all time points. huAR9.6–Gd–DTPA facilitated contrast enhancement in mice tumors 3 h after conjugate administration compared to the pre-injection image (*p* = 0.0198, Figure 4d). Interestingly, the effect on contrast was lost after this 3 h time point as the T1 relaxation times returned to baseline within the tumors (Figure 4d). No contrast enhancement was observed compared to the pre-injection MR scans for tumors in the mice treated with the huIgG–Gd–DTPA isotype control conjugate (Figure 4d).

**Figure 5:**
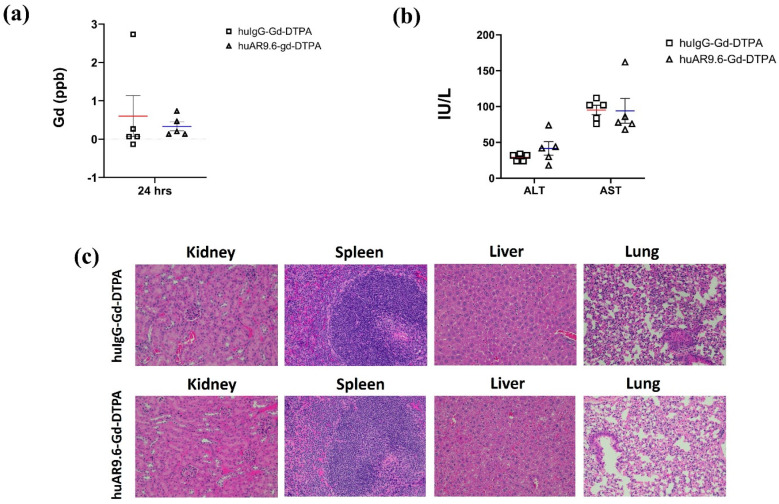
Toxicity characterization of antibody Gd acetate. (**a**) Gd contents in serum of mice injected with huIgG–Gd–DTPA and huAR9.6–Gd–DTPA 24 h post-intravenous (tail vein) administration (*n* = 5/group); (**b**) biochemical analysis of liver enzymes ALT and AST in mouse serum samples in huIgG–Gd–DTPA and huAR9.6–Gd–DTPA groups 24 h post-intravenous (tail vein) administration (*n* = 5/group); (**c**) H&E staining of kidney, spleen, liver and lung tissues to check morphological changes due to Gd toxicity (20× magnification).

Biochemical and histopathological analyses were performed to determine potential Gd-induced toxicity to vital organs. ICP–MS conducted on serum collected 24 h post-conjugate administration revealed that there was no toxic accumulation of free Gd (<0.5 ppb) or Gd conjugates in the systemic circulation in the huAR9.6–Gd–DTPA group (Figure 5a). Liver enzymes ALT (alanine transaminase) and AST (aspartate transaminase) were in the normal range in both huAR9.6–Gd–DTPA and huIgG–Gd–DTPA groups (Figure 5b). Normal untreated mice had ALT and AST ranges of 25–60 IU/L and 50–100 IU/L, respectively [31]. ALT levels in huIgG–Gd–DTPA and huAR9.6–Gd–DTPA were 29.2 and 41.6, respectively, whereas the AST levels were 95.2, and histological assessment revealed no abnormal morphological changes in the kidneys, spleen, liver, or lung tissues from mice that received the huAR9.6 and huIgG conjugates (Figure 5c). These studies indicate that the huAR9.6–Gd–DTPA conjugate provides contrast enhancement for detecting orthotopic tumors within the pancreas in athymic nude mice and does not cause treatment-induced toxicity.

## 4. Discussion

Tumor imaging has a crucial role in the diagnosis of aggressive cancers like PDAC, as it can aid in the determination of cancer stage and in the assessment of surgical resection margins [32]. The most widely used imaging for PDAC includes MRI, computed tomography (CT), and endoscopic ultrasonography (EUS) [33,34]. Early detection is a challenging and unmet need in the field of pancreatic cancer research, and it could provide insights into the significance of early treatment and its influence on survival outcomes in patients with PDAC and other pancreatic cancers. MRI has been well established as a robust tool for the high-spatial-resolution imaging of cancerous lesions, and it is more applicable than many other imaging technologies for detecting small lesions (less than 2 cm) [6,35]. In the recent literature, several groups have investigated the utility of MRI in detecting pancreatic cancer. A scoring system based on multiparametric MRI, incorporating gadolinium-enhanced imaging, provides better resolution for the diagnosis and staging of chronic pancreatitis by assessing features such as pancreatic duct irregularities, parenchymal structure, and fibrotic changes in the organ [36]. Another group reported that a gadolinium-based contrast agent targeting fibronectin potentiates combined MRI and optical imaging at low doses, allowing for the highly sensitive and specific monitoring of chemotherapy effectiveness in PDAC [37].

In this study, we combine the spatial resolution advantage provided by MRI contrast imaging with a humanized targeted anti-MUC16 antibody to detect MUC16-expressing lesions of pancreatic cancer in mouse models. We showed that huAR9.6–Gd–DTPA successfully and consistently detected pancreatic tumors in the subcutaneous model, where tumor burden was low, and tumors were starting to be palpable through the skin. In the early tumor burden setting, huAR9.6–Gd–DTPA enhanced contrast to a much higher degree 24 h after conjugate administration than the non-targeted huIgG counterpart. In an anatomically relevant orthotopic model of pancreatic cancer, huAR9.6–Gd–DTPA was able to detect tumor burden and margins (highlighted by region of interests circles in Figure 4) at 3 h post-conjugate administration at a higher resolution/contrast than the huIgG control, further substantiating the utility of the novel agent in detecting lesions of pancreatic cancer in both the early stages and in anatomically relevant pancreatic tumor.

As the designed conjugate is particular to MUC16 expressed in pancreatic cancer, it restricts its distribution and/or prolonged exposure to other tissues, thereby mitigating any off-target toxicity and false-positive images of normal tissue proximal to the pancreas. Additionally, this novel conjugate can be used in magnetic resonance cholangiopancreatography (MRCP), a unique form of MRI that creates a 2D image of the vasculature involved, allows for anatomically accurate reconstruction of the pancreas, gall bladder, bile duct and the liver, and aids in the detection of abnormalities in the anatomically and clinically relevant sites that could be potentially affected in a patient with MUC16-expressing pancreatic cancer [4,38].

The conventional MRI protocol comprising the qualitative (non-quantitative) method has a disadvantage in detecting small tumors. We performed dynamic T1 mapping to produce quantitative maps with which to overcome qualitative MRI constraints. A previous study showed that the T1 quantitative mapping of relaxation time (ms) facilitates accurate and sensitive cancer diagnosis [39]. The reduced T1 relaxation time following huAR9.6–Gd–DTPA administration was evident from the contrast changes compared to the pre-injection conditions. Our study demonstrated that the generated T1 maps were sensitive to the administered Gd concentration at 100 nmol/gram of body weight.

Additionally, unconjugated gadolinium chelates are minuscule in molecular size and can rapidly diffuse back into the vasculature and out of the tissue. Interestingly, gadolinium chelates conjugated to monoclonal antibodies result in the increased accumulation of such a contrast agent in the tissue, eventually reducing the tissue-specific relaxation time. The enhanced permeability and retention (EPR) effect is partly what governs such tumor imaging outcomes, facilitates the accumulation of macromolecular structures, like a heavy monoclonal antibody conjugated to gadolinium, and facilitates “passive targeting” for the field for drug delivery to solid tumors like PDAC [40]. However, it is becoming increasingly evident that not every tumor is identical. Like the tumor proper, the vasculature can be heterogeneous as it is influenced by many components, including but not limited to inflammation, the presence of macrophages (high in PDAC), the secretion of angiogenic factors, and the stromal collagen architecture [41,42].

It has been reported that the successful conjugation ratio of Gd/mAb should be less than 37 (mol/mol) [43]. Our study reports that the ratio of Gd/huAR9.6 for the orthotopic model was 31.32 (mol/mol). Additionally, the stability of the antibody–Gd–DTPA contrast complex depends on the pH. Strong acidic conditions cause lower stability of the conjugate. Our results depict that the conjugate storage pH was between 7 and 8, which indeed increases its stability.

This study does come with its limitations. For this antibody–MRI probe to be effectively used in clinical settings, detailed studies on its pharmacokinetics and biodistribution in preclinical murine models with sufficient power will need to be performed, followed by clinically meaningful assessments for biodistribution in humans. These studies should be conducted to determine the optimal imaging window. Gadolinium-induced contrast is usually acquired minutes after the administration of the contrast agent, which is not what we report in this study. As the addition of the antibody to this MRI probe makes it a “heavier” entity, its biodistribution will be significantly changed. Additionally, safety and efficacy studies need to be conducted in humans, mainly to understand if it can be used in the context of kidney disease, as these patients are vulnerable to gadolinium-related adverse events [44,45].

While preclinical studies often rely on high-field MRI scanners (6 T or 7 T) for superior resolution, translating this MUC16-targeted MRI probe to the more widely available 3 T clinical scanners is achievable, despite its lower resolution, with careful optimization of the probe design and imaging protocols. Additionally, tools like perfusion MRI are downstream applications of this probe that have not been studied herein. Pharmacokinetic studies using this probe in the right models can aid in our understanding of the usability of this agent in the context of the rapid imaging requirements of perfusion MRI protocols [46,47].

## 5. Conclusions

In conclusion, we report on the design and development of a novel MUC16-targeted antibody MRI probe to diagnose MUC16-expressing pancreatic cancer in the early stages with smaller lesions and under aggressive disease burden with sizable tumors. This study awas conducted to bring a humanized antibody to the clinic and use it in a “theranostic” sense for the high-resolution detection of MUC16-expressing pancreatic cancer lesions in patients.

## Data Availability

The original contributions presented in this study are included in the article. Further inquiries can be directed to the corresponding author(s).
